# Outdoor Growth Characterization of an Unknown Microalga Screened from Contaminated* Chlorella* Culture

**DOI:** 10.1155/2017/5681617

**Published:** 2017-03-05

**Authors:** Shuhao Huo, Changhua Shang, Zhongming Wang, Weizheng Zhou, Fengjie Cui, Feifei Zhu, Zhenhong Yuan, Renjie Dong

**Affiliations:** ^1^School of Food and Biological Engineering, Jiangsu University, Zhenjiang 212013, China; ^2^Guangzhou Institute of Energy Conversion, Chinese Academy of Sciences, Guangzhou 510640, China; ^3^College of Engineering, China Agricultural University, Beijing 100083, China; ^4^Institute of New Energy and New Materials, South China Agricultural University, Guangzhou, China

## Abstract

Outdoor microalgae cultivation process is threatened by many issues, such as pest pollution and complex, changeable weather. Therefore, it is difficult to have identical growth rate for the microalgae cells and to keep their continuous growth. Outdoor cultivation requires the algae strains not only to have a strong ability to accumulate oil, but also to adapt to the complicated external environment. Using 18S rRNA technology, one wild strain* Scenedesmus* sp. FS was isolated and identified from the culture of* Chlorella zofingiensis*. Upon contamination by* Scenedesmus* sp., the species could quickly replace* Chlorella zofingiensis *G1 and occupy ecological niche in the outdoor column photobioreactors. The results indicated that* Scenedesmus* sp. FS showed high alkali resistance. It also showed that even under the condition of a low inoculum rate (OD_680_, 0.08),* Scenedesmus* sp. FS could still grow in the outdoor raceway pond under a high alkaline environment. Even under unoptimized conditions, the oil content of* Scenedesmus* sp. FS could reach more than 22% and C16–C18 content could reach up to 79.68%, showing that this species has the potential for the biodiesel production in the near future.

## 1. Introduction

Due to the exorbitant cost input into nutritive salts such as chemical fertilizers and high energy consumption in microalgae harvest, the microalgal biodiesel has not yet been successfully applied in commercial production [1–4]. Utilizing sunlight to magnify the cultivation of microalgae under outdoor conditions is an effective way to reduce the cost of microalgal cultivation. The current studies on energy microalgae are mainly carried out at indoor labs as there are many difficulties in outdoor cultivation [5–7]. In outdoor cultivation, the microalga of interest is often vulnerable to contamination with viruses, bacteria, fungi, insect pupae, rotifers, protozoa, or other unwanted algal species. Among them, rotifers and protozoa are the two organisms that are able to seize ecosystem niche quickly due to their small body, simple structure, and fast reproduction speed. They prey on microalgae cells, resulting in a great reduction of microalgae cell concentration, and exceedingly threatening the microalgae production. Meanwhile, the complex and changeable outdoor weather conditions make the cultivation of microalgae have an uneven growth rate and thus production is often difficult to be carried out [8–10].

**Figure 1:**
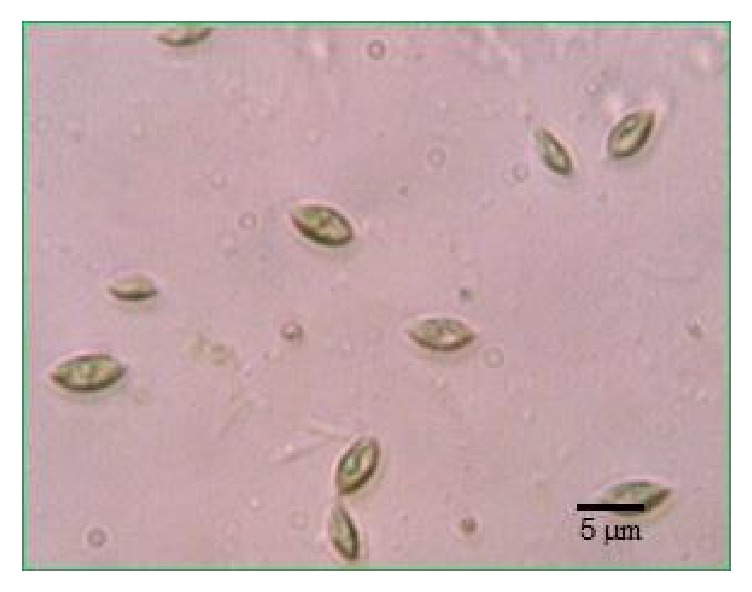
The wild microalgal strain in the outdoor pond observed with light microscope at ×400 magnification.

Excellent microalgal strain is crucial to the realization of microalgal biodiesel production. Outdoor cultivation requires the selected algal strain not only to have a strong ability to accumulate oil but also to adapt to the external environment. The lipid accumulation of microalgae can be improved by changing the element content in the culture medium such as the deprivation of nitrogen, phosphorus, or other elements, while the outdoor adaptability of microalgae is more difficult to be improved in a short time. Through the amplification of 18S rRNA sequences of unknown algal strains, the wild microalgal strain screened from contaminated* Chlorella zofingiensis *G1 in an outdoor column photobioreactor was preliminarily identified in this paper.* Chlorella zofingiensis* G1 could be quickly replaced by the wild microalgal strain (Figure 1), which could occupy ecological niche quickly, showing high alkali resistance and antipollution performances. The wild microalgal fronds are unicellular ellipse in shape with a smooth surface, and the cell size is approximately 5.0–7.0 *μ*m in length and 2.0–4.0 *μ*m in width under light microscope. At the same time, this paper also examined the possibility for large-scale cultivation in the outdoor raceway pond.

## 2. Materials and Methods

### 2.1. Materials

Colonies of microalgae were isolated from contaminated* Chlorella zofingiensis* G1 in an outdoor photobioreactor in the district of Sanshui, Foshan city, China (23°03′N–112°09′E). The isolated algal cells were cultured and maintained in a BG11 medium [11] at 25°C under continuous illumination by cool-white fluorescent lamps (light intensity: 2000 lux) in a 500 mL Erlenmeyer flask. Aeration and mixing were achieved by the sparging air with 6.0% CO_2_ through a glass-filter, which was inserted to the bottom of the reactor and the flow rate of gas was 0.5 vvm, regulated by the gas flow meter (Model G, Aalborg Instruments & Controls, Inc., Orangeburg, NY, USA). The temperature of the culture media was 25 ± 1°C, regulated by the room air conditioner (Gree Electric Appliances Inc., Zhuhai, Guangdong, China). After 6 days of cultivation, when the cells were in the logarithmic phase, the cultures were used for outdoor experiments.

A 40 L vertical tubular outdoor photobioreactor (8.7 cm × 160 cm = diameter × height) was used to cultivate the above-mentioned strain as seed cultures for the outdoor raceway pond amplification cultivation.

rTaq, pMD18–T, and T4 DNA Ligase were obtained from Takara Biotech Co., Ltd., China. EasyPure Quick Gel Extraction Kit was obtained from Beijing TransGen Biotech Co., Ltd., China. The nucleotide sequences of these primers (Table 1) were synthesized by Sangon Biotech Co., Ltd., China. DNA sequencing was analyzed by Shanghai Life Technologies Corporation, China. The primers NS1 and NS8 were used to clone the 18S rRNA sequence of microalgae. The primers M13 (−40) Forward and M13 Reverse were used to clone the inserted gene fragment in pMD18-T and confirm the success of TA cloning.

### 2.2. Identification Methods

#### 2.2.1. Microscopic Observation

After shaking evenly, a drop of 0.05 mL microalgae sample was dripped onto the slide, and the sample was covered by glass (18 × 24 mm) and observed with polarizing microscope from Nikon Instruments Eclipse LV100 POL at ×400 magnification.

#### 2.2.2. Isolation of Genomic DNA of the Wild Microalgal Strain

Wild microalgal strain was harvested during logarithmic phase after 3-4 days of cultivation in a BG11 medium, frozen in liquid nitrogen, and grounded using a pestle and mortar. The genomic DNA was isolated by the CTAB method [12].

#### 2.2.3. PCR Amplification of 18S rRNA from the Wild Microalgal Strain

The 18S rRNA was amplified by PCR using the NS1 and NS8 universal primers as shown in Table 1 and the genomic DNA was used as a template for PCR amplification. PCR amplification was carried out in 0.2 mL tubes. The PCR mixture included 10x PCR Buffer (Mg^2+^ plus) 5 *μ*L, dNTPs (2.5 mM) 4 *μ*L, NS1 (20 *μ*M) 1 *μ*L, NS8 (20 *μ*M) 1 *μ*L, genomic DNA 1 *μ*L, rTaq (5 U/*μ*L) 0.2 *μ*L, and deionized water 37.8 *μ*L, with a total volume of 50 *μ*L. Amplification conditions were as follows: 30 cycles at 94°C for 30 s, 50°C for 30 s, and 72°C for 2 min, followed by a final extension at 72°C for 5 min. PCR products were fractionated in 2% (w/v) agarose gels and stained with ethidium bromide.

#### 2.2.4. The Purification, Ligation, and Transformation of PCR Product

The PCR product was recovered using EasyPure Quick Gel Extraction Kit (Trans, Beijing), according to the instruction book. The amplification products were ligated into pMD18-T vector (Takara) and then transformed and sequenced, according to standard procedures described by Sambrook et al. [13].

#### 2.2.5. The Identification of Positive Transformants

Amplified fragments with resistance to ampicillin were picked from the medium, they were cultured in 3 mL LB liquid medium at 150 rpm for about 20 h, and the colony was identified through PCR using 1 *μ*L culture. Transformed* E. coli *DH5*α* were picked from the medium containing 100 *μ*g/mL ampicillin. The PCR mixture included 10x PCR Buffer (Mg^2+^ plus) 5 *μ*L, dNTPs (2.5 mM) 4 *μ*L, M13 (−40) Forward (20 *μ*M) 1 *μ*L, M13 Reverse (20 *μ*M) 1 *μ*L, liquid culture 1 *μ*L, rTaq (5 U/*μ*L) 0.2 *μ*L, and deionized water 37.8 *μ*L, with a total volume of 50 *μ*L. Amplification conditions were as follows: 30 cycles at 94°C for 30 s, 46°C for 30 s, and 72°C for 2 min, followed by a final extension at 72°C for 5 min. PCR products were size fractionated in 2% (w/v) agarose gels and stained with ethidium bromide. Volume of 1.5 mL culture was selected for sequencing analysis (Life Technology, Shanghai).

#### 2.2.6. Lipid Content and Fatty Acid Composition Analysis

Bigogno's method (2002) was applied to quantify the amount of total lipid content [14]. Fatty acid composition analysis was carried out by the saponification reaction with the participation of base catalyst [15].

### 2.3. Outdoor Raceway Pond Cultivation of the Wild Microalgae

**Figure 2:**
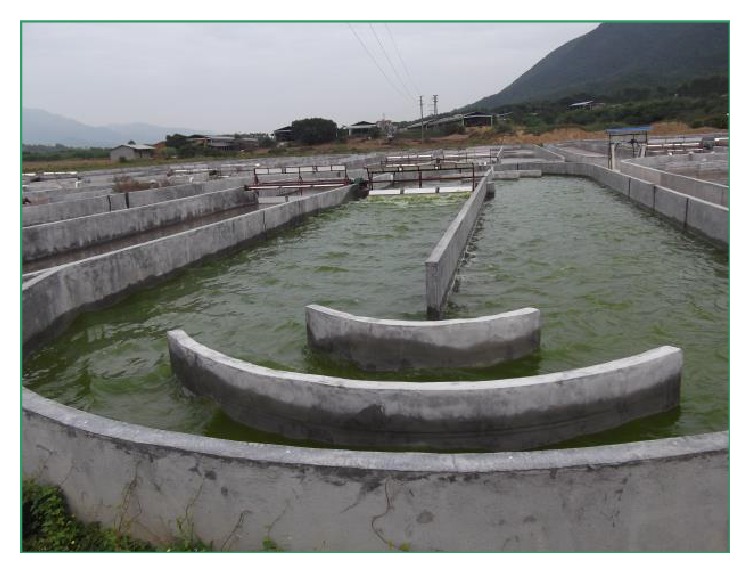
Photograph of outdoor ponds for wild microalgae cultivation (8 m × 50 m).

The outdoor raceway ponds (brick cement ponds) were located in Sanshui district, Foshan city, China (23°03′N–112°09′E). The ponds were 50 cm in height, 8 m in width, and 50 m in length from north to south with semicircular arc at both ends and had average of 23–25 cm depth of water (Figure 2). Paddle wheel device was installed to circulate the pool liquid, which was operated at about 10 cm depth and rotated at the speed of 15 rpm. Cultivation water was mountain spring water (Table 2). Nutrient composition was specified in Table 2. A large amount of chemical fertilizer was added to the outdoor raceway pond, including 300 mg/L of CO (NH_2_)_2_, 60 mg/L of KH_2_PO_4_, and 60 mg/L of MgSO_4_. The initial optical density (OD_680_) of the culture was controlled between 0.3 and 0.5 when appropriate amount preculture broths were inoculated into the 25 m long small pond during the logarithmic phase. In the process of cultivation, the pH value was not adjusted. The pH, temperature, and light intensity were recorded 4 times a day (8:00, 11:00, 14:00, and 17:00). The biomass concentration was determined by measuring the OD_680_ value of the sample in the pond at 17:00. After 7 days of cultivation, the wild microalgae were harvested.

## 3. Results and Discussion

### 3.1. Wild Algae Identification Using 18S rDNA Technology

**Figure 3:**
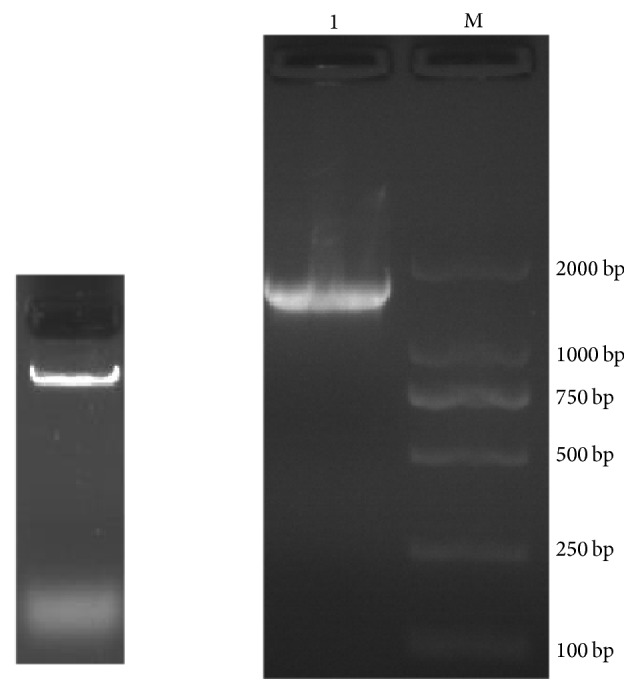
Genomic DNA from wild microalgal strains (left) and amplification results of 18S rRNA of wild microalgal strain (1: 18S rRNA band; M: DL2000 DNA marker) (right).

The CTAB method was used to extract the genomic DNA of wild microalgae strains. The results in Figure 3 showed that the genomic DNA sample was complete and no degradation phenomenon was found in 1% (w/v) agarose gels electrophoresis stained with ethidium bromide.

**Figure 4:**
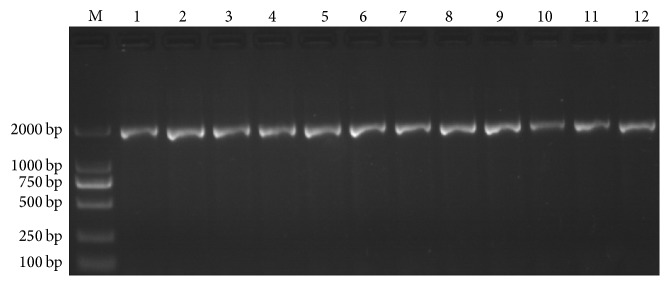
Electropherogram of PCR products from 12 positive transformants, carrying pMD18–T in which the 18S rRNA gene sequence of the wild alga was cloned. M13 primers were used for identification.

By using universal primers NS1 and NS8 and the wild algae genomic DNA as a template for PCR amplification, a 1767 bp band was obtained, as shown in Figure 3. A total of 12 positive transformants of* E. coli* DH5*α* were identified using primers M13 (−40) Forward and M13 Reverse using 1 *μ*L culture as template. Electropherogram of PCR products was from 12 positive transformants of* E. coli* DH5*α* containing recombinant pMD18-T. The bands were identified by electrophoresis. As shown in Figure 4, the results suggested that all 18S rRNA fragments with approximate length of 2 kb were successfully connected to the pMD18-T vector. The 12 positive transformants were named as 18S-1~18S-12. Of them, 18S-1~18S-5 were selected for sequencing and the results were completely consistent (analyzed by Shanghai Life Biotech Co., Ltd.).

**Figure 5:**
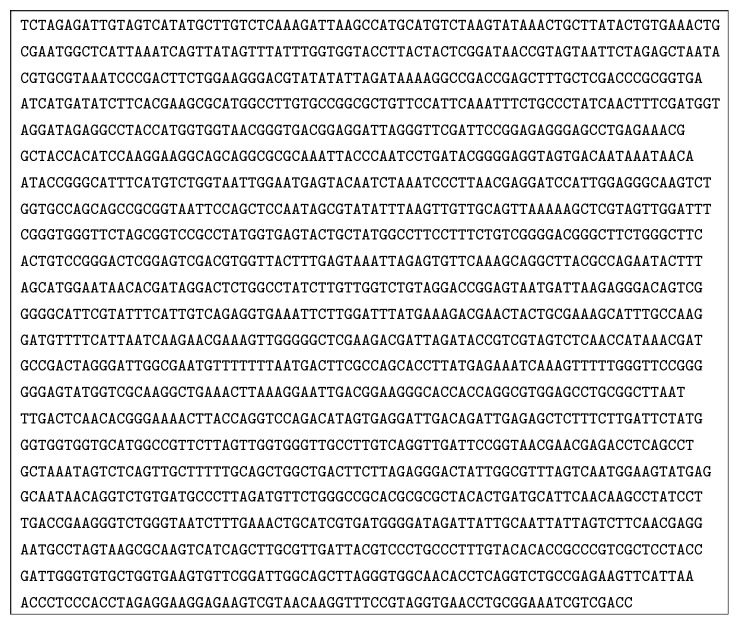
18S rDNA gene sequence of the wild strain.

**Figure 6:**
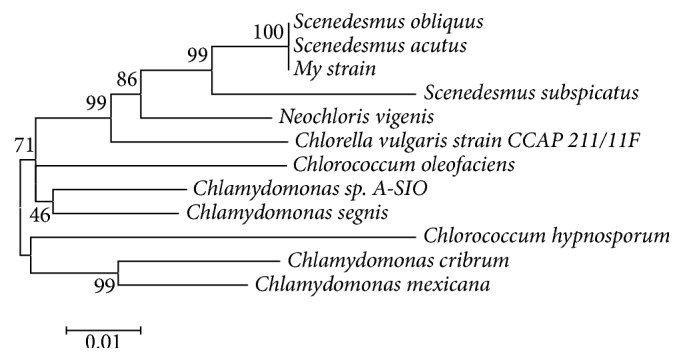
Phylogenetic tree constructed based on the 18S rRNA gene sequences of 11 strains of green algae and the experimental microalga (Bootstrap values are indicated as percentages at the nodes).

The 18S rRNA gene sequence amplified from this strain is 1767 bp in length (Figure 5), which showed similarities with other known sequences from green algae based on the BLAST* n* results, with homology above 99% to* Scenedesmus obliquus *and* Scenedesmus acutus*. The phylogenetic analysis indicated that this strain has a close relationship with* Scenedesmus* sp., named* Scenedesmus* sp. FS (Figure 6). The sequences of 18S rRNA gene (fragment) of those microalgae in NCBI GenBank were as follows:* Scenedesmus obliquus*: FR865738.1;* Scenedesmus acutus*: AJ249512.1;* Scenedesmus subspicatus*: AJ249514.1;* Neochloris vigenis*: M74496.1;* Chlorella vulgaris* strain CCAP 211/11F: AY591515.1;* Chlorococcum oleofaciens*: KM020101.1;* Chlamydomonas* sp. A-SIO: AF517100.1;* Chlamydomonas segnis*: U70593.1;* Chlorococcum hypnosporum*: U41173.1;* Chlamydomonas cribrum*: LC086333.1;* Chlamydomonas mexicana*: AF395434.1;* Scenedesmus* sp. FS: KY268297.

### 3.2. Cultivation of* Scenedesmus *sp. FS in the Outdoor Raceway Pond

**Figure 7:**
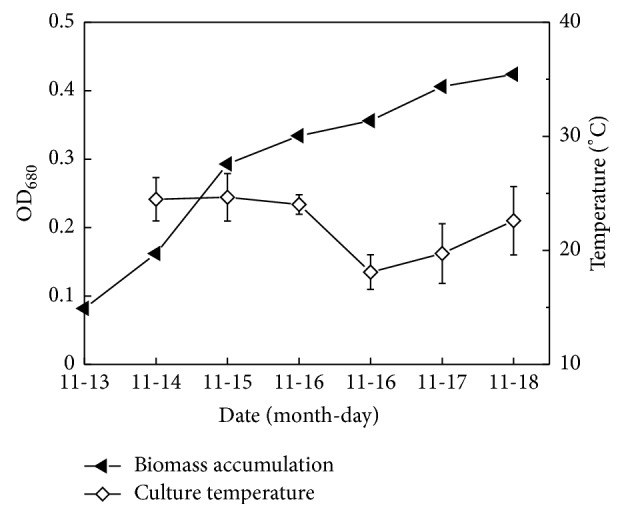
Growth curve and fluctuation of culture temperature (an average value from 8:00 am to 5:00 pm each day).

As shown in Figure 7, the optical density of inoculum was very low (OD_680_, 0.08), and the raceway pond had a large surface area to volume ratio, which could inhibit the growth of single cells at the initial phase due to strong light. But in fact* Scenedesmus* sp. FS was still able to adapt to the environment of outdoor raceway pond. The growth and metabolism of* Scenedesmus *sp. FS was active, and the algae did not experience the lag phase and directly got into the logarithmic phase with a rapid growth rate.

**Figure 8:**
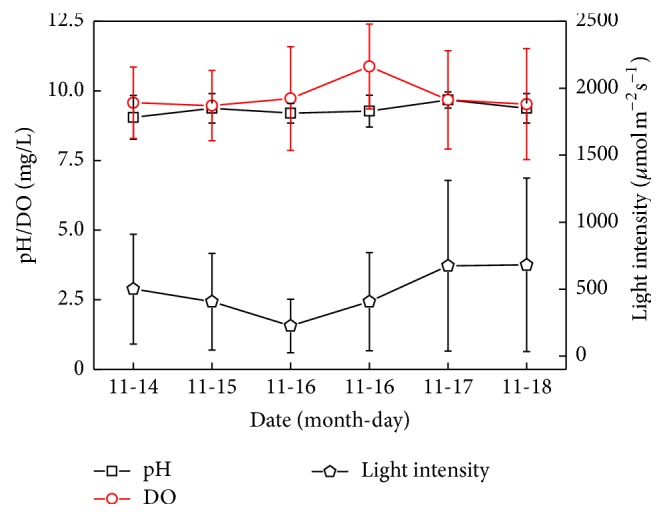
The fluctuation of light intensity, pH, and dissolved oxygen (DO) (the average value in the range of 8:00 am~5:00 pm) under outdoor conditions.

In the experiment, light intensity and temperature in the outdoor changed obviously, and the highest light intensity reached more than 1500 *μ*mol/(m^2^·s), and water temperature ranged from 18.1 to 24.7°C. Sánchez et al. (2008) reported that the optimal growth temperature of* Scenedesmus almeriensis* was 35°C, and it could still survive at 48°C. This algae could tolerate high light intensity (1625 *μ*mol/(m^2^·s)) and could also accumulate 0.55% (wt%) lutein under this light intensity [16]. In this experiment, the wild algae* Scenedesmus* sp. FS was also able to tolerate similar light intensity. Additionally, the wild alga was able to grow under high alkaline condition with pH values between 9.0 and 9.7 (Figure 8). The dissolved oxygen changed between 9.5 and 10.9 mg/L daily, which was relatively stable. It can be seen that the wild algae* Scenedesmus *sp. FS could well adapt to the outdoor conditions in the process of cultivation. The pest pollution and other alien invasive algae in this experiment were not significant, which indicates the scale-up potential of the wild algae under outdoor culture conditions. In future studies, outdoor cultivation conditions need further optimization to obtain larger biomass and higher oil yield rate.

### 3.3. Oil Content and Fatty Acid Composition of* Scenedesmus* sp. FS

Numerous studies have shown that nitrogen deficiency can promote accumulation of oil in microalgae [17]. Goldberg and Cohen found that phosphorus deficiency could also significantly promote oil accumulation of* Monodus subterraneus* [18]. Under these conditions, the fixed CO_2_ of algal cells would be converted with priority into lipid or carbohydrates, rather than proteins [8]. In normal culture condition, fatty acid synthesized was mainly used in the synthesis of membrane sugar-based glyceride and phospholipids, which accounted for about 5–20% of the dry weight of the algae cells. Under the nitrogen deficiency and other adverse environmental conditions, algae cell would change the oil synthesis route, beginning to accumulate neutral fat of about 20–50% of the dry weight, with triacylglycerol (TAG) as the main component. After 6 days of cultivation, the total nitrogen and phosphorus concentration in the pond were 70.8 ± 2.8 mg/L and 6.0 ± 0.1 mg/L, respectively, indicating a sufficient supply of nitrogen and phosphorus. Oil content in the algal cells increased slowly and oil content of* Scenedesmus* sp. FS was not high with fatty acid composition 22.0 ± 1.9% of the algal weight.

The compositions of fatty acid methyl esters (FAMEs) were shown in Table 3. The microalgal lipids mainly contained FAMEs with 16–18 carbons with a high cetane number. These lipids show better fuel properties and low temperature performance and were considered suitable for sustainable biodiesel production [19]. The content of FAMEs C16–C18 in the wild strain* Scenedesmus* sp. FS reached 79.68%, with C16:0 24.55% and C18:2 40.64%, showing good potential for development of biodiesel.

## 4. Conclusion

Using 18 s rDNA molecular technology, the dominant microalgae strain screened from contaminated* Chlorella zofingiensis* G1 in an outdoor photobioreactor was identified. The isolated species belonged to* Scenedesmus *genus, which was named* Scenedesmus *sp. FS. Under the conditions of a low concentration inoculation, this strain still had good alkali resistance and robust adaption to the stress of the outdoor environment. It had great potential as a large-scale cultivation strain for biodiesel production. Further research would be focused on the optimization of the culture conditions and the use of random mutagenesis technology, directed evolution method, or other technical means to obtain higher biomass and oil yield of the alga* Scenedesmus *sp. FS.

## Figures and Tables

**Table 1:** Oligonucleotide primers used in this work.

Primer	Sequence (5′→ 3′)
NS1	GTAGTCATATGCTTGTCTC
NS8	TCCGCAGGTTCACCTACGGA
M13 (−40) Forward	GTTTTCCCAGTCACGAC
M13 Reverse	CAGGAAACAGCTATGAC

**Table 2:** The quality of mountain spring water^*∗*^.

Elements	Content (*μ*g/L)
Mn	5.99 ± 3.46
Fe	22.37 ± 17.52
P	128.28 ± 1.50
Si	3481.00 ± 9.90
Na	28.27 ± 0.64
K	160.05 ± 6.12
Mg	1174.60 ± 9.33
Zn	26.84 ± 4.02
Ca	1979.60 ± 12.16
Mo	n. d.
Cu	n. d.

^*∗*^Note: average values of water samples collected in two different locations; n.d.: not detected.

**Table 3:** Fatty acid composition (wt%) of the wild *Scenedesmus *sp. FS cells.

Fatty acid	wt%
C16:0	24.55
C16:1	2.74
C18:0	2.56
C18:2	40.64
C18:3	9.19
C20:1	4.64
C20:2	4.53
C22:1	4.29
C24:1	2.45
